# Seroprevalence of SARS-CoV-2 in Niger State: Pilot Cross-Sectional Study

**DOI:** 10.2196/29587

**Published:** 2023-10-17

**Authors:** Hussaini Majiya, Mohammed Aliyu-Paiko, Vincent Tochukwu Balogu, Dickson Achimugu Musa, Ibrahim Maikudi Salihu, Abdullahi Abubakar Kawu, Ishaku Yakubu Bashir, Aishat Rabiu Sani, John Baba, Amina Tako Muhammad, Fatimah Ladidi Jibril, Ezekiel Bala, Nuhu George Obaje, Yahaya Badeggi Aliyu, Ramatu Gogo Muhammad, Hadiza Mohammed, Usman Naji Gimba, Abduljelili Uthman, Hadiza Muhammad Liman, Sule Alfa Alhaji, Joseph Kolo James, Muhammad Muhammad Makusidi, Mohammed Danasabe Isah, Ibrahim Abdullahi, Umar Ndagi, Bala Waziri, Chindo Ibrahim Bisallah, Naomi John Dadi-Mamud, Kolo Ibrahim, Abu Kasim Adamu

**Affiliations:** 1 Department of Microbiology, Ibrahim Badamasi Babangida University, Lapai, Nigeria; 2 Center for Applied Sciences and Technology Research, Ibrahim Badamasi Babangida University, Lapai, Nigeria; 3 Trans-Saharan Disease Research Center, Ibrahim Badamasi Babangida University, Lapai, Nigeria; 4 Department of Biochemistry, Ibrahim Badamasi Babangida University, Lapai, Nigeria; 5 Department of Biology, Ibrahim Badamasi Babangida University, Lapai, Nigeria; 6 Department of Computer Science, Ibrahim Badamasi Babangida University, Lapai, Nigeria; 7 Department of Geography, Ibrahim Badamasi Babangida University, Lapai, Nigeria; 8 Department of Mathematics, Ibrahim Badamasi Babangida University, Lapai, Nigeria; 9 General Hospital, Minna, Nigeria; 10 Niger State Ministry of Health, Minna, Nigeria; 11 Ibrahim Badamasi Babangida Specialised Hospital, Minna, Nigeria

**Keywords:** COVID-19, pandemic, SARS-CoV-2, seroprevalence, serology, epidemiology, Niger State, Nigeria, COVID-19 testing, social distancing

## Abstract

**Background:**

The COVID-19 pandemic caused by SARS-CoV-2 is causing ongoing human and socioeconomic losses.

**Objective:**

To know how far the virus has spread in Niger State, Nigeria, a pilot study was carried out to determine the SARS-CoV-2 seroprevalence, patterns, dynamics, and risk factors in the state.

**Methods:**

A cross-sectional study design and clustered, stratified random sampling strategy were used to select 185 test participants across the state. SARS-CoV-2 IgG and IgM rapid test kits (colloidal gold immunochromatography lateral flow system) were used to determine the presence or absence of antibodies to the virus in the blood of sampled participants across Niger State from June 26 to 30, 2020. The test kits were validated using the blood samples of some of the Nigeria Center for Disease Control–confirmed positive and negative COVID-19 cases in the state. SARS-CoV-2 IgG and IgM test results were entered into the Epi Info questionnaire administered simultaneously with each test. Epi Info was then used to calculate the arithmetic mean and percentage, odds ratio, *χ*^2^ statistic, and regression at a 95% CI of the data generated.

**Results:**

The seroprevalence of SARS-CoV-2 in Niger State was found to be 25.4% (47/185) and 2.2% (4/185) for the positive IgG and IgM results, respectively. Seroprevalence among age groups, genders, and occupations varied widely. The COVID-19 asymptomatic rate in the state was found to be 46.8% (22/47). The risk analyses showed that the chances of infection are almost the same for both urban and rural dwellers in the state. However, health care workers, those who experienced flulike symptoms, and those who had contact with a person who traveled out of Nigeria in the last 6 months (February to June 2020) were at double the risk of being infected with the virus. More than half (101/185, 54.6%) of the participants in this study did not practice social distancing at any time since the pandemic started. Participants’ knowledge, attitudes, and practices regarding COVID-19 are also discussed.

**Conclusions:**

The observed Niger State SARS-CoV-2 seroprevalence and infection patterns meansuggest that the virus has widely spread, far more SARS-CoV-2 infections have occurred than the reported cases, and there is a high asymptomatic COVID-19 rate across the state.

## Introduction

The COVID-19 pandemic was caused by a novel coronavirus (SARS-CoV-2) that is believed to have crossed from bats to humans for the first time [1-3]. COVID-19 is an infectious disease of the respiratory system of humans and animals, and the virus can be transmitted through facial openings including the mouth, nostrils, and (maybe) eyes [2-4].

The first case of COVID-19 in Niger State, Nigeria, was announced by the Nigeria Center for Disease Control (NCDC) on April 10, 2020; this was about 6 weeks after the first confirmed case (index case) of COVID-19 in Nigeria was announced on February 27, 2020, when a foreigner in Lagos tested positive for SARS-CoV-2. Since then, many cases have been confirmed in the state, and this number is still increasing [5]. As part of the measures to curtail the spread of SARS-CoV-2, strict lockdown (restricting people to their homes except for essential needs, eg, medicine and food) was enforced in the state from March 25 to June 9, 2020. However, full compliance to the strict lockdown by the citizens of the state may not have been achieved or possible due to socioeconomic and cultural reasons, disbelief, and conspiracy theories. Many people would have to go out on a daily basis to work and provide for their families, and markets are usually open spaces bustling with large crowds of people. Many people did not believe in COVID-19, especially the highly contagious nature of the disease. There are also no efficient and robust housing and biometric data management systems where everyone is accounted for, especially for the purposes of employment, health, security, and social welfare. If these are available, foods and other goods purchased online can be sent to houses with ease. Additionally, utilities are not provided or are inadequately supplied in most cases. It is difficult for people to stay at home and comply with the strict lockdown in such situations. After the lockdown was eased, there has been enhanced enforcement of the compulsory use of face masks in public places and adherence to physical distancing in the state [5].

Niger State is one of the 36 states in Nigeria, with Minna being its capital. It has 25 local government areas that are fairly distributed among the three geopolitical zones of the state in terms of land mass and population. In terms of land mass, Niger State is the largest state (76,363 km^2^) in Nigeria and has the 18th-highest (5,556,247 people) population [6,7]. However, as of December 21, 2020, Niger State is ranked 28th among the states in COVID-19 cases reported in Nigeria. The total number of reported COVID-19 cases in the state as of December 21, 2020, is 381, with 12 deaths, while for Nigeria (with a population of about 206,630,269), there have been 79,789 COVID-19 cases and 1231 deaths [5-8]. It is generally believed that the reported COVID-19 cases in the state and Nigeria are far below the actual SARS-CoV-2 infections in the populations. This may be due to polymerase chain reaction (PCR)–based SARS-CoV-2 test limitations in many states of Nigeria and unknown proportions of mild or asymptomatic COVID-19 cases that may not be diagnosed or reported. The presence and detection of antibodies to SARS-CoV-2 in the blood samples of participants likely indicate that they were infected at some point since the start of the pandemic. Therefore, serologic assays can be used to determine population-based estimates of SARS-CoV-2 infection, including those who had a mild or asymptomatic infection or who were never tested despite symptoms [9-11].

For COVID-19, like most infectious diseases, the isolation of the etiologic agent SARS-CoV-2 through the tissue or cell plate culture technique would be the gold standard method for the diagnostic test. However, plate culturing is usually laborious, time-consuming, complex, and costly, and is impossible to use, especially for epidemiological studies where large samples may be involved. Additionally, even though reverse transcriptase–PCR has been predominantly used to test for the agent of COVID-19 worldwide, including in Nigeria, it is also laborious, time-consuming, costly, and complex [12-14].

Infection by many pathogens including viruses does elicit the production of antibodies in humans and animals even if no symptoms manifested. The detection of the antibodies in the whole blood/serum/plasma of humans and animals has been used for preliminary diagnoses of infectious diseases [9-15]. Additionally, because of the relative ease of use and simplicity of the antigens and antibody test kits compared to cell/tissue cultures and PCR, they are mostly used in epidemiological studies to determine infectious disease prevalence, patterns, dynamics, and risk factors [12-15]. Antigen and antibody test kits, unlike other methods, can detect previous exposure to infectious agents [9-15]. This information is important, especially for COVID-19, which has an assumed high rate of asymptomatic cases, to see how far the virus has spread, infection patterns, and the effectiveness of the enforced social distancing measures. This pilot study was aimed at determining the SARS-CoV-2 seroprevalence, patterns, dynamics, and risk factors for contracting COVID-19 in Niger State, Nigeria. It was also aimed at assessing the knowledge, attitudes, and practices of people regarding COVID-19 and related control measures in the state.

## Methods

### Study Design and Population

A cross-sectional study design and clustered, stratified random sampling strategy were used.

**Figure 1.**
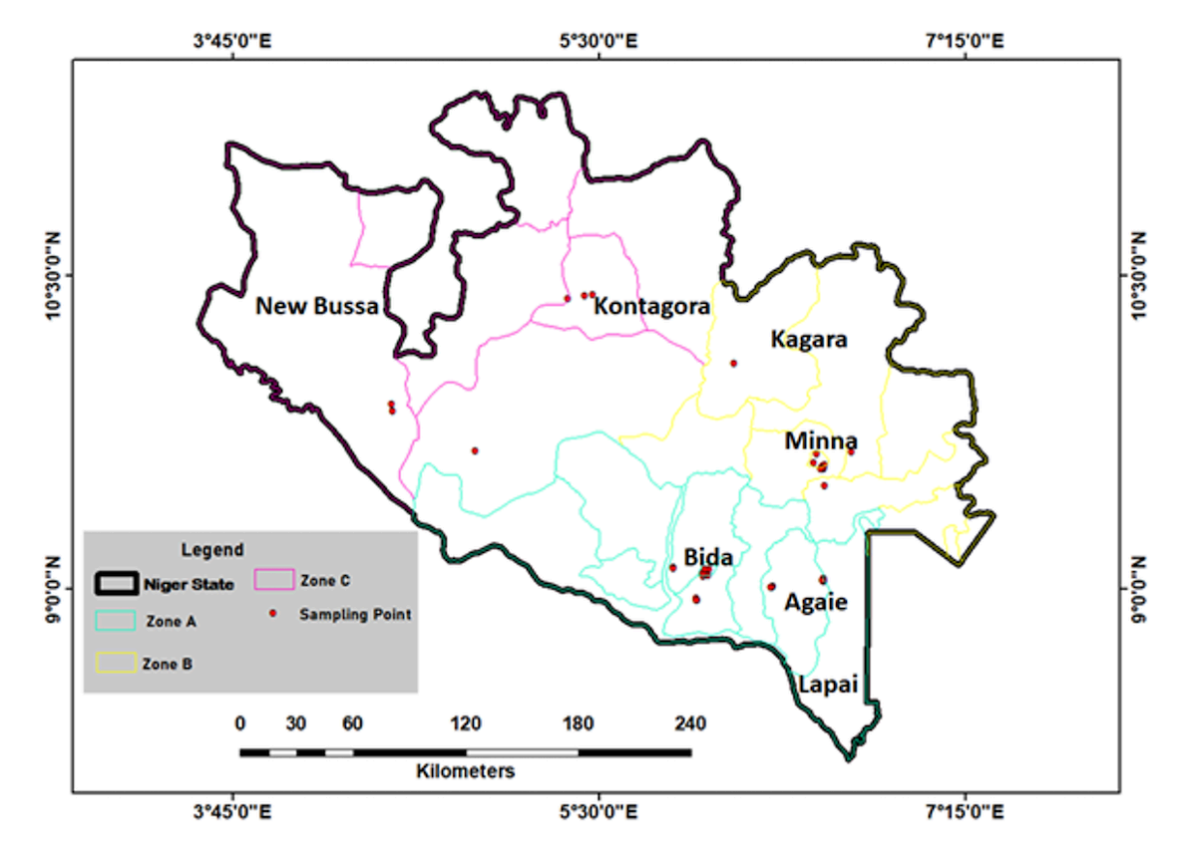
Map of Niger State showing the sites where samples were taken, tests carried out, and questionnaires administered in the three geopolitical zones (zone A, zone B, and zone C) of the state for the pilot SARS-CoV-2 seroprevalence study carried out from June 26 to 30, 2020, in Niger State, Nigeria. The exact names, latitudes, and longitudes of the sampling points can be found in Multimedia Appendix 1.

The study area was Niger State, and its residents were the study population (Figure 1). Niger State is one of the federating geopolitical states in Nigeria; its capital is Minna. Other major towns in the state are Bida, Kontagora, Suleja, New Bussa, Mokwa, Lapai, and Agaie. The three geopolitical zones (zone A, zone B, and zone C) in the state were covered (Figure 1). Place of residence (classified as urban or rural), gender, occupation (classified as health care worker or non–health care worker), and age group/range were the stratifications that were applied in the places chosen in each zone (Figure 1). The selected sampling points (Multimedia Appendix 1) were socioeconomic areas, such as hospitals and primary health care centers, motor parks, markets, village/community heads’ households, sawmills, and schools. Considering the stratifications, people in the selected sampling points were randomly approached and recruited to participate in the study. OpenEpi Toolkit (Dean AG, Sullivan KM, Soe MM) was used to calculate the minimum sample size for the study. Since this is a pilot study and SARS-CoV-2 prevalence is not known in Niger State, which has a population of about 5 million people, we assumed that the overall SARS-CoV-2 prevalence would be about 50%, with 95% confidence in the estimate, 10% absolute precision, and a 1.0 design effect (for random cluster surveys); therefore, the minimum required sample size was 97 participants. A total of 185 participants were enrolled in this study. Among individuals approached for recruitment, the average acceptance/participation rate was 87.3% (185/212). From June 26 to 30, 2020, and with full consent to participate in the study, samples were taken randomly from 185 participants (with almost equal distribution among the three geopolitical zones) for SARS-CoV-2 IgG and IgM rapid tests, and the questionnaire (created by Epi Info 7.2.2.6; Centers for Disease Control and Prevention) was administered simultaneously.

### Ethics Approval

Ethical approval for this study was given by the Research Ethics Committee of the Niger State Ministry of Health (STA/495/Vol/152). Consent was also sought from each of the participants prior to the test and questionnaire administration, and only individuals who gave full consent were included in the study. Parents/guardians were responsible for the consent of their wards who participated in the study and were younger than 18 years.

### Specimen Type and SARS-CoV-2 IgG and IgM Rapid Test

SARS-CoV-2 IgG and IgM rapid tests were carried out using the whole blood of the participants. The test is a qualitative membrane-based immunoassay for the detection of COVID-19 antibodies in whole blood. The tests were carried out and interpreted according to the kit manufacturer’s instructions. The test result of each participant was recorded and entered into the Epi Info questionnaire administered for that particular participant. The SARS-CoV-2 IgG and IgM rapid test kits were validated with the blood samples of those individuals that were confirmed by the NCDC through PCR as positive or negative for COVID-19 in Niger State. All 10 NCDC-confirmed positive cases, tested positive for the IgG for SARS-CoV-2, while the 5 NCDC-confirmed negative individuals (that had never tested positive before) tested negative for the IgG and IgM for SARS-CoV-2. This means that 100% sensitivity and specificity were observed for the test kits used in the study.

### Epi Info Questionnaire and Statistics

To be able to determine the COVID-19 prevalence, patterns, dynamics, and risk factors for contracting the disease in Niger State, a questionnaire (Multimedia Appendix 2) was designed and created using Epi Info 7.2.2.6. The questionnaire was designed to ask questions with categorical responses (yes or no) and to accommodate the test results of the participants; this allowed 2 × 2 statistics tables to be created and used for calculating the SARS-CoV-2 infection odds ratios and linear regression (multivariate analysis) for many scenarios. SARS-CoV-2 IgG and IgM test results were entered into the Epi Info questionnaire administered simultaneously with each test. Epi Info was used to calculate the arithmetic mean and percentage, odds ratio, *χ*^2^, and regression at 95% CI of the data generated. Demographics; SARS-CoV-2 prevalence and COVID-19 asymptomatic rate; and the knowledge, attitudes, and practices of the participants were expressed as percentages. For the risk of contracting SARS-CoV-2 analyses (odds ratio and *χ*^2^), the SARS-CoV-2 IgG status (prevalence) was the dependent variable, while the demographics and risks were the independent variables.

## Results

### Demographics; Knowledge, Attitudes, and Practices; Travel History; and Flulike Symptoms of the Participants

The demographic characteristics; knowledge, attitudes, and practices; travel history; and flulike symptoms of the participants in this study are shown in Table 1. More than half (n=101, 54.6%) of the 185 participants in this study had not practiced social distancing at any time since the pandemic started (January to June 2020), even as the lockdown was enforced in the state. The majority (n=114, 61.6%) of the participants practiced hand and face hygiene. Almost all (n=181, 97.8%) of the participants had not traveled out of Nigeria since the beginning of the year 2020 when the pandemic started. Only a few (n=4, 2.2%) of the participants traveled out of Nigeria in the last 6 months (January to June 2020) and returned. However, 24 (13%) participants had contact with someone who traveled out of the country in the last 6 months (January to June 2020). The majority (n=113, 61.1%) of the participants did not experience any flulike symptoms since when the pandemic started (January 2020 to June 2020). Only 72 (38.9%) participants experienced flulike symptoms (January to June 2020).

**Table 1.** Demographics; knowledge, attitudes, and practices; travel history; and flulike symptoms of the participants in a pilot SARS-CoV-2 seroprevalence study carried out from June 26 to 30, 2020, in Niger State, Nigeria.

Variable		Participants, n (%)	Exact 95% CI (%)	
**Age (years; n=185)**				
	≤5	15 (8.1)	4.61-13.02	
	6-17	26 (14.1)	9.39-19.91	
	18-29	34 (18.4)	13.08-24.72	
	30-41	45 (24.3)	18.33-31.16	
	42-53	37 (20.0)	14.49-26.50	
	54-65	20 (10.8)	6.73-16.20	
	≥66	8 (4.3)	1.89-8.34	
**Gender (n=185)**				
	Male	103 (55.7)	48.21-62.96	
	Female	82 (44.3)	37.04-51.79	
**Resident (n=185)**				
	Urban	115 (62.2)	54.75-69.17	
	Rural	70 (37.8)	30.83-45.25	
**Occupation (n=185)**				
	Health care workers	43 (23.2)	17.36-30.00	
	Non–health care workers	142 (76.8)	70.00-82.64	
**Health care workers: gender (n=43)**				
	Male	22 (51.2)	35.46-66.69	
	Female	21 (48.8)	33.31-64.54	
**Knowledge (n=185)**				
	Aware of COVID-19	151 (81.6)	75.28-86.92	
	Not aware of COVID-19	34 (18.4)	13.08-24.72	
**Belief (n=185)**				
	COVID-19 is in Niger State	109 (58.9)	51.46-66.08	
	COVID-19 is not in Niger State	76 (41.1)	33.92-48.54	
**Hand and face hygiene (n=185)** ^**a**^				
	Yes	114 (61.6)	54.20-68.66	
	No	71 (38.4)	31.34-45.80	
**Social distancing (n=185)** ^**a**^				
	Yes	84 (45.4)	38.09-52.87	
	No	101 (54.6)	47.13-61.91	
**Travel history (n=185)** ^**a**^				
	Traveled overseas	4 (2.16)	0.59-5.44	
	Did not travel overseas	181 (97.84)	94.56-99.41	
**Contact history (n=185)** ^**a**^				
	Contact with overseas returnee	24 (12.97)	8.49-18.69	
	No contact with overseas returnee	161 (87.03)	81.31-91.51	
**Flulike symptoms (n=185)** ^**a**^				
	Experienced flulike symptoms	72 (38.92)	31.85-46.35	
	Did not experience flulike symptoms	113 (61.08)	53.65-68.15	

a Variables were for the period of 6 months (January to June 2020) prior to the study being conducted.

### SARS-CoV-2 Seroprevalence, COVID-19 Asymptomatic Rate, and Infection Risks in Niger State

The SARS-CoV-2 seroprevalence for the 185 participants in Niger State was 25.4% (n=47) and 2.2% (n=4) for the positive IgG and IgM tests, respectively, as of June 26-30, 2020 (Table 2). The number of participants that did not experience flulike symptoms in the last 6 months (January to June 2020) and tested positive for SARS-CoV-2 IgG amounted to the COVID-19 complete asymptomatic rate in Niger State. The COVID-19 asymptomatic rate in the state was found to be 47% (22/47). SARS-CoV-2 seroprevalence among age groups, gender, and occupations varied widely. Among age groups, the SARS-CoV-2 seroprevalence was found to be 33.3% (15/45) for those 30-41 years, 32.4% (12/37) for those 42-53 years, 30% (6/20) for those 54-65 years, 25% (2/8) for those ≥66 years, 19.2% (5/26) for those 6-17 years, 17.7% (6/34) for those 18-29 years, and 6.7% (1/15) for those ≤5 years. A seroprevalence of 27.2% (28/103) was recorded for male participants and 23.2% (19/82) for female participants in the state. A SARS-CoV-2 seroprevalence of 37.2% (16/43) was recorded for health care workers in Niger State. Among the non–health care workers in the state, the SARS-CoV-2 seroprevalence recorded was 21.8% (31/142). The SARS-CoV-2 seroprevalence among the urban dwellers in the state stood at 27.8% (32/115), while for the rural dwellers, it was 21.4% (15/70). The same SARS-CoV-2 seroprevalence (1/4, 25%) was recorded among the overseas returnees and those that did not travel 25.41% (46/181). However, a higher SAR-CoV-2 seroprevalence (10/24, 41.7%) was recorded for those who had contact with the overseas returnees compared to those who did not have contact with the returnees (37/161, 23%).

**Table 2.** SARS-CoV-2 seroprevalence, infection risks, and COVID-19 asymptomatic rate as of June 26-30, 2020, in Niger State, Nigeria.

Variable		SARS-CoV-2 seropositivity for IgG (n=47), n (%)	SARS-CoV-2 seroprevalence, n/N (%)	Exact 95%		Positive COVID-19 IgG test			
						2 × 2 statistics (univariate analysis)		Linear regression (multivariate analysis)	
						Odds ratio	*P* value (*χ*^2^)	Coefficient	*P* value
Overall (IgG)		47 (100)	47/185 (25.4)	19.30-32.31		—^a^	—	—	—
Overall (IgM; n=4)		4 (100)	4/185 (2.2)	0.59-5.44		—	—	—	—
**Age (years)**				—		—	—	—	—
	≤5	1 (2.1)	1/15 (6.7)						
	6-17	5 (10.6)	5/26 (19.2)						
	18-29	6 (12.8)	6/34 (17.7)						
	30-41	15 (31.9)	15/45 (33.3)						
	42-53	12 (25.5)	12/37 (32.4)						
	54-65	6 (12.8)	6/20 (30.0)						
	≥66	2 (4.3)	2/8 (25.0)						
**Gender**				—		0.81	.65	0.00	.96
	Male	28 (60.0)	28/103 (27.2)						
	Female	19 (40.4)	19/82 (23.2)						
**Resident**				—		1.41	.43	0.00	.97
	Urban	32 (68.1)	32/115 (27.8)						
	Rural	15 (31.9)	15/70 (21.4)						
**Occupation**				—		2.21	.07	0.01	.91
	Health care workers	16 (34.0)	16/43 (37.2)						
	Non–health care workers	31 (66.0)	31/142 (21.8)						
**Knowledge**				—		—	—	—	—
	Aware of COVID-19	43 (91.5)	43/151 (28.5)						
	Not aware of COVID-19	4 (8.5)	4/34 (11.8)						
**Belief** **s**				—		—	—	—	—
	COVID-19 is in Niger State	37 (78.7)	37/109 (33.9)						
	COVID-19 is not in Niger State	10 (21.3)	10/76 (13.2)						
**Behavior (A)**				—		1.90	.12	–0.07	.50
	Hand and face hygiene	34 (72.3)	34/114 (29.8)						
	No hand and face hygiene	13 (27.7)	13/71 (18.3)						
**Behavior (B)**				—		2.16	.04^b^	0.10	.31
	Social distancing	28 (59.6)	28/84 (33.3)						
	No social distancing	19 (40.4)	19/101 (18.8)						
**Travel history**				—		0.98	>.99	–0.19	.41
	Traveled overseas	1 (2.1)	1/4 (25.0)						
	Did not travel overseas	46 (97.9)	46/181 (25.4)						
**Contact history**				—		2.39	.09	0.16	.15
	Contact with overseas returnee	10 (21.3)	10/24 (41.7)						
	No contact with overseas returnee	37 (78.7)	37/161 (23.0)						
**Flulike symptoms**				—		2.20	.03^b^	0.14	.04^b^
	Experienced flulike symptoms	25 (53.2)	25/72 (34.7)						
	Did not experience flulike symptoms	22 (46.8)	22/113 (19.5)						

a Not applicable.

b Values are significantly different (*P*=.05).

To determine the risk factors of SARS-CoV-2 infection and the effectiveness of COVID-19 preventive measures enforced in the state, 2 × 2 statistics tables were used to calculate the odds ratios for many scenarios. When the gender of the participants and positive COVID-19 IgG results were cross-tabulated, the risk ratio recorded for female participants was 0.85 (Table 2).

The risk analyses showed that the chances of infection are almost the same for both urban and rural dwellers in the state even though COVID-19 seroprevalence among urban dwellers was a little higher than that of rural dwellers. Health care workers, those who experienced flulike symptoms, and those that had contact with a person that traveled out of Nigeria in the last 6 months (January to June 2020) are at double the risk of being infected with the virus. However, in linear regression multivariate analysis, only “experienced flu-like symptoms” was significant at 95% CI among them. The risk analyses showed that returning from overseas did not confer protection or pose any increased risk of contracting the virus (Table 2).

## Discussion

### Key Findings

This SARS-CoV-2 seroprevalence pilot study was carried out to understand how far the virus has spread in Niger State, Nigeria, and to determine the patterns, dynamics, and risk factors of COVID-19 in the state. We used a cross-sectional study design and clustered, stratified random sampling strategy to select 185 study participants across three geopolitical zones of the state; this was an effort to have a fair representation of the state even though the sample size was small.

The life expectancy in Nigeria is currently 55.8 years [8]. The gender of the participants reflected the ratio of males to females in Nigeria, which is currently 50.6% males to 49.4% females (Table 1) [7]. In Nigeria, currently, 52% of the population lives in urban areas, while 48% are in rural areas [8].

Before COVID-19 vaccines became available, other ways of preventing the transmission of SARS-CoV-2 (the causative agent of the COVID-19 pandemic) among humans are social/physical distancing measures and good sanitation and hygiene practices. Adherence to these COVID-19 preventive measures will be impacted by the knowledge and beliefs of people about the disease since the measures involve some behavioral changes and practices. People can only believe what they know (or are aware of) and can only practice when they believe.

There are many reasons why people did not observe social distancing (Table 1). The first is poverty. The level of poverty in society is high, and many people have to go out on a daily basis to work to feed their families. Markets are usually open spaces bustling with large crowds, and most transactions are done with the physical exchange of cash, which prevents people from social distancing. Additionally, poverty causes people to gather in places where food and money are distributed and where physical distancing and other required COVID-19 control measures may not be observed or enforced [13].

The second reason is the prevalence of disbelief, myths, and conspiracy theories. Many people did not believe in COVID-19 (Table 1), especially regarding the highly contagious nature of the disease. This may be the chief reason why many people did not care to observe social/physical distancing (Table 1) when not enforced on them at ATMs, markets, religious gatherings, motor parks, shops, supermarkets, etc. Additionally, myths and conspiracy theories, such as COVID-19 not affecting Black people, high environmental temperature and weather killing off the virus, or COVID-19 being for rich people and elites, are some of the reasons why people are slow to accept the enormity of the pandemic and, therefore, take observance of social and physical distancing measures lightly [13].

Third, there are no efficient and robust housing and biometric data management systems where everyone is accounted for, especially for the purposes of employment, health, security, and social welfare. If these are available, foods and other goods purchased online can be sent to houses with ease. In addition, utilities such as power, water, or internet are not provided or are inadequately supplied in most cases. It is difficult for people to stay at home and observe social/physical distancing in such situations [13].

Participants in this study were asked whether they traveled out of Nigeria or had contact with someone that traveled out of Nigeria since the pandemic started (last 6 months; January-June 2020). The first confirmed case (index case) of COVID-19 in Nigeria was announced on February 27, 2020, when a foreigner in Lagos tested positive for SARS-CoV-2. Soon after, many people, including the contacts of the index case and those who returned to the country and their contacts, tested positive for the virus. Although overseas travel prior to the border closures and lockdowns in Nigeria was associated with the increased chance/risk of contracting COVID-19, this might have changed over time due to more community transmission of the virus (Table 2).

Looking at the dynamics and trajectory of COVID-19 in Nigeria in the early days of the pandemic when COVID-19 cases were reported already in urban areas in Nigeria, it was supposed to take a few weeks before the virus reached rural areas [5]. Since preventive measures such as social/physical distancing (lockdown) and use of face masks were enforced in these early days in most states of Nigeria including the Niger State, living in the rural areas of Niger State and other states of Nigeria ought to have been a protective factor against COVID-19 if the preventive measures were strictly observed. More than half (101/185, 54.6%) of the participants in this study did not practice social distancing (Table 1) at any time since the pandemic started, even as the lockdown was enforced in the state; this may be the reason why the risk of infection of the virus was the same for the urban and rural dwellers, who may be less observant of the preventive measures (Table 2). It is also an indication of community spread of SARS-CoV-2 in Niger State.

Additionally, the participants were asked whether they have had flulike experiences in the last 6 months (January to June 2020) since the COVID-19 index case was announced in Nigeria; this helped to deduce the COVID-19 asymptomatic rate in Niger State, which was 47% (22/47) (Table 2). Other SARS-CoV-2 serosurveys worldwide reported similar high asymptomatic rates of COVID-19 [16-18]. It has been reported that the majority of people infected with SARS-CoV-2 (about 50%-75%) are usually asymptomatic [19,20].

The seroprevalence of SARS-CoV-2 in Niger State was found to be 25.4% (47/185) and 2.2% (4/185) for positive IgG and IgM results, respectively. The observed seroprevalence was higher than in most of the SARS-CoV-2 serosurveys carried out around the same time in other parts of the world [17,18,21], and only 1 study in India reported a higher seroprevalence of 54.1% [22]. However, considering the 25.4% (47/185) SARS-CoV-2 seroprevalence observed in Niger State and the reported COVID-19 cases for Niger State and Nigeria as of June 30, 2020 (when this study was conducted) and December 21, 2020, SARS-CoV-2 infections occurred far more than the reported cases in the state and Nigeria [5]. Our data suggest that there are over 5000 times more SARS-CoV-2 infections than the number of reported cases in Niger State and over 900 times more SARS-CoV-2 infections than the number of reported cases in Nigeria. The high SARS-CoV-2 seroprevalence observed in this study may be due to many factors including a high COVID-19 asymptomatic rate and the lack of social distancing adherence in the state as observed in this study. An unknown high proportion of asymptomatic COVID-19 cases may not be diagnosed or reported, so our observed SARS-CoV-2 seroprevalence in the state will be more reliable and closer to the true prevalence of the disease than the official reported cases. As of December 21, 2020, and based on the reported COVID-19 cases and deaths, the fatality rates for COVID-19 in Niger State and Nigeria stood at 3.15% and 1.54%, respectively [5]. However, when the observed 25.4% (47/185) SARS-CoV-2 seroprevalence was considered, the fatality rates drastically reduced to 0.0009% and 0.024% for Niger State and Nigeria, respectively.

Usually, IgM becomes detectable in the whole blood/serum/plasma of patients 2-3 days from the onset of COVID-19 symptoms or after 10 days in cases of asymptomatic COVID-19 [10,15]. The IgM level in the blood peaks after 14 days of the SARS-CoV-2 infection and disappears after 28 days of infection [10,15]. However, IgG production starts after 14 days of infection and remains in the blood for long-term immunity [10,15]. The timeline for production and disappearance of IgG and IgM are useful in the interpretation of the COVID-19 IgG and IgM rapid test. The test kit detecting only IgM means that the participant/patient is at the early stage of the infection, while the kit detecting only IgM means that the participant/patient had a past infection and recovered. However, the test kits detecting both the IgG and IgM at the same time means the participant/patient may be in the recovery stage of the infection. In this study, IgG and IgG plus IgM were observed. This means that the overwhelming majority of the participants who tested positive (positive IgG only) on the tests had past infections and recovered (Table 2). Only a few patients tested positive for both IgG and IgM and recovering from the infection (Table 2).

The SARS-CoV-2 seroprevalence among the age groups and gender correlated with the most mobile/active of the age groups and gender in our society (Table 2). The age groups 30-41 years, 42-53 years, and 54-65 years were the most mobile of the age groups, while men were more mobile than women and could, therefore, contract the virus easily. We observed less likely odds of contracting COVID-19 among females compared to males (Table 2). This means that being a female is a protective factor against the infection of SARS-CoV-2 in Niger State. This also correlated with the COVID-19 seroprevalence recorded among male and female participants (Table 2). The lower risk of infection for females in this study may be due to physical attributes such as less mobility and activity compared to males in society. Generally, the case fatality of COVID-19 varied widely worldwide (1%-20%), with more cases and fatalities observed in males compared to females [23,24].

High SARS-CoV-2 seroprevalence (16/43, 37%) and doubled odds of contracting COVID-19 among health care workers (Table 2) were observed. It is expected that health care workers have a higher COVID-19 prevalence compared to non–health care workers because they are the frontline workers responsible for the diagnosis, treatment, and management of patients, including those with symptomatic and asymptomatic COVID-19 [25-27]. These enormous essential tasks for controlling the COVID-19 pandemic together with the inadequate or lack of personal protective equipment in some instances and the high asymptomatic rate of COVID-19 among people put health care workers at greater risk of contracting and transmitting the disease. The double odds of being positive for SARS-CoV-2 were also observed for the participants who experienced flulike symptoms and observed social distancing since the pandemic started (January to June 2020; Table 2). The double odds for flulike symptoms were expected and in line with our findings that about 50% of the SARS-CoV-2 infections in the state were asymptomatic (Table 2). However, the double odds for observing social distancing are not correct and may be due to confounding issues; this was confirmed in the linear regression multivariate analysis (Table 2).

### Limitations

The study has some limitations. First, selection bias may exist as it was more difficult to recruit participants who were 5 years or younger. Second, the sample size used in this pilot study and the number of SARS-CoV-2 rapid test kits used for validation were small. The SARS-CoV-2 rapid test kits that are suitable for epidemiological studies are costly; this limits the sample size of this pilot study. Third, to get a quick understanding of the levels of knowledge, attitudes, and practices about COVID-19, we kept the questionnaire short and simple, which might have limited the depth of the study.

### Conclusions

To the best of our knowledge, this study is the first pilot SARS-CoV-2 seroprevalence data for Nigeria. The study revealed SARS-CoV-2 seroprevalence, patterns, dynamics, and risk factors in Niger State, Nigeria. The seroprevalence of SARS-CoV-2 in Niger State was found to be 25.41% (47/185) and 2.16% (4/185) for the positive IgG and IgM test results, respectively. Seroprevalence among age groups, genders, and occupations varied widely due to the differences in mobility and activity as well as the occupational exposures and hazards. The COVID-19 asymptomatic rate in the state was found to be 46.8% (22/47). The risk analyses showed that the chances of infection are almost the same for both urban and rural dwellers in the state. However, health care workers, those that experienced flulike symptoms, and those that had contact with a person that traveled out of Nigeria in the last 6 months have a doubled risk of being infected with the virus. More than half (101/185, 54.59%) of the participants in this study did not practice social distancing at any time since the pandemic started. The observed Niger State SARS-CoV-2 seroprevalence and infection patterns mean that the virus has widely spread, more SARS-CoV-2 infections have occurred than have been reported, and there is a high asymptomatic COVID-19 rate across the state. Our data suggest that >5000 times more SARS-CoV-2 infections occurred than the number of reported cases in Niger State and >900 times more than the number of reported cases in Nigeria.

## Supplementary material

10.2196/29587 Multimedia Appendix 1 Names, latitudes, and longitudes of the of the sampling and testing points.

10.2196/29587 Multimedia Appendix 2 Epi Info questionnaire administered simultaneously with the SARS-CoV-2 rapid IgG/IgM test to each of the participants in the study.

## Abbreviations

NCDC: Nigeria Center for Disease Control
PCR: polymerase chain reaction
