# Supplementary material for: Seroprevalence of SARS-CoV-2 in Niger State: Pilot Cross-Sectional Study
Source: JMIRx Med. 2023 Oct 17;4:e29587. doi: 10.2196/29587 (PMC10595504; doi:10.2196/29587)
Supplement: Multimedia Appendix 1 [file xmed-v4-e29587-s001.pdf]

Appendix 1

Names, latitude, and longitude of the of the sampling and testing points

S/No.	Northing	Easting	Sampling and Testing Location	Zone
1	9.038590	6.570100	Emir's Palace Lapai	A
2	9.052600	6.571000	General Hospital Lapai	A
3	9.040500	6.574600	Malle Road Lapai	A
4	9.037750	6.573640	Katsina Road Lapai	A
5	9.041600	6.567800	Jantabo Road Lapa	A
6	9.044600	6.567100	Ahmadu Bello Road Lapai	A
7	9.013600	6.328400	General Hospital Agaie	A
8	9.011100	6.326510	Amir's Palace Agaie	A
9	9.011200	6.325900	GRA Agaie	A
10	9.010720	6.321380	Ghana Area Agaie	A
11	9.096700	6.024400	General Hospital Agaie	A
12	9.074900	5.992400	Federal Medical Center Bida	A
13	9.103400	5.852300	Edozhgi Village	A
14	9.102000	5.852900	PHC Edozhigi	A
15	9.063810	5.994700	Ramatu Dangana Bida	A
16	9.068120	6.017920	Banyagi Area Bida	A
17	9.071470	6.002400	Government College Bida	A
18	9.096190	6.007650	Lemu Road Bida	A
19	8.946000	5.965700	Doko/Buku Village	A
20	8.955600	5.963500	Doko Town	A
21	8.945500	5.963200	Vunchi	A
22	9.578170	6.570390	IBBU Guest House Minna	B
23	9.603611	6.526111	Limawa Minna	B
24	9.576366	6.571456	Shango Minna	B
25	9.588717	6.565747	Abdulsalam Garage	B
26	9.657623	6.705674	Shakwatu	B
27	9.596470	6.575980	MI Wushishi	B
28	9.495190	6.579170	Kuchi, Paiko	B
29	9.581389	6.566944	City Gate Minna	B
30	9.573611	6.556111	Brighter School	B
31	9.645278	6.538611	Peogeot Automobile Bahago Bosso	B
32	9.853250	4.508514	Hydro Hotel New Bussa	C
33	10.080330	6.142510	Tegina Village	C
34	10.403068	5.429310	Behind First Bank Kontagora	C
35	10.407263	5.469689	General Hospita Kontagora	C
36	10.387652	5.346697	Jega Village along Kontagora Jega Road	C
37	9.887250	4.507427	Burgu LGA Secretariate New Bussa	C
38	9.661493	4.903878	Ibbi Community New Bussa	C
