# Supplementary material for: Seroprevalence of SARS-CoV-2 in Niger State: Pilot Cross-Sectional Study
Source: JMIRx Med. 2023 Oct 17;4:e29587. doi: 10.2196/29587 (PMC10595504; doi:10.2196/29587)
Supplement: Multimedia Appendix 2 [file xmed-v4-e29587-s002.pdf]

Appendix 2

EPIINFO Questionnaire administered simultaneously with the SARS CoV-2 Rapid IgG/IgM Test to each of the participants in the study

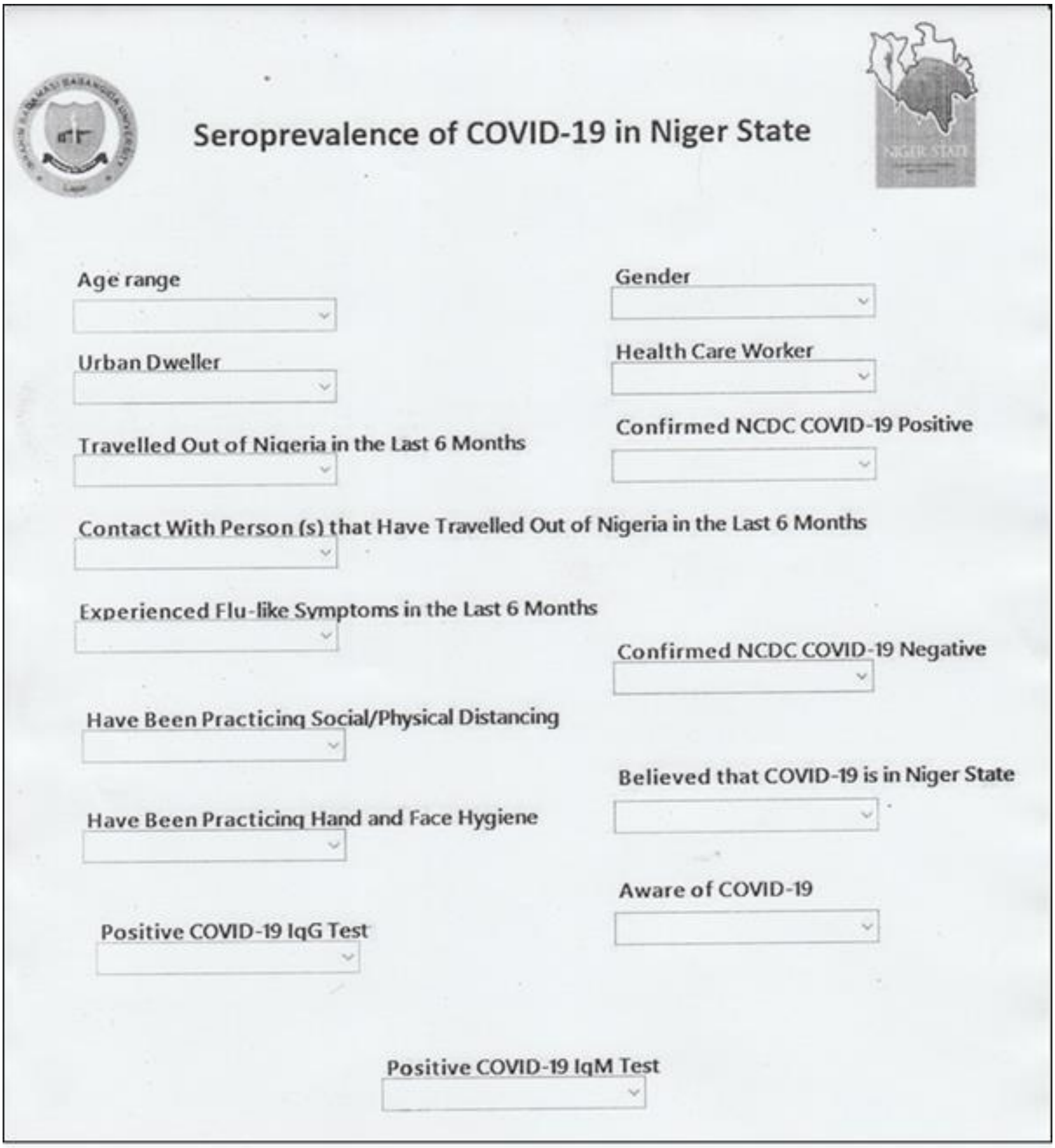

Seroprevalence of COVID-19 in Niger State

Age range

Gender

Urban Dweller

Health Care Worker

Travelled Out of Nigeria in the Last 6 Months

Confirmed NCDC COVID-19 Positive

Contact With Person (s) that Have Travelled Out of Nigeria in the Last 6 Months

Experienced Flu-like Symptoms in the Last 6 Months

Confirmed NCDC COVID-19 Negative

Have Been Practicing Social/Physical Distancing

Believed that COVID-19 is in Niger State

Have Been Practicing Hand and Face Hygiene

Aware of COVID-19

Positive COVID-19 IgG Test

Positive COVID-19 IgM Test
